# The eIF4E homolog 4EHP (eIF4E2) regulates hippocampal long-term depression and impacts social behavior

**DOI:** 10.1186/s13229-020-00394-7

**Published:** 2020-11-23

**Authors:** Shane Wiebe, Xiang Qi Meng, Sung-Hoon Kim, Xu Zhang, Jean-Claude Lacaille, Argel Aguilar-Valles, Nahum Sonenberg

**Affiliations:** 1 Department of Biochemistry, McGill University, McIntyre Medical Building, 3655 Promenade Sir William Osler, Montreal, QC H3G 1Y6 Canada; 2 Department of Biochemistry, Goodman Cancer Research Centre, McGill University, 1160 Pine Avenue West, Room 614, Montreal, QC H3A 1A3 Canada; 3 Department of Neuroscience and CIRCA, University of Montreal, Succ. Downtown, PO Box 6128, Montreal, QC H3C 3J7 Canada; 4 Department of Neuroscience, Carleton University, Health Sciences Building, 1125 Colonel by Drive, Ottawa, ON K1S 5B6 Canada

**Keywords:** 4EHP, GIGYF2, Long-term depression, Social behavior, Animal models

## Abstract

**Background:**

The regulation of protein synthesis is a critical step in gene expression, and its dysfunction is implicated in autism spectrum disorder (ASD). The eIF4E homologous protein (4EHP, also termed eIF4E2) binds to the mRNA 5′ cap to repress translation. The stability of 4EHP is maintained through physical interaction with GRB10 interacting GYF protein 2 (GIGYF2). Gene-disruptive mutations in *GIGYF2* are linked to ASD, but causality is lacking. We hypothesized that *GIGYF2* mutations cause ASD by disrupting 4EHP function.

**Methods:**

Since homozygous deletion of either gene is lethal, we generated a cell-type-specific knockout model where *Eif4e2* (the gene encoding 4EHP) is deleted in excitatory neurons of the forebrain (4EHP-eKO). In this model, we investigated ASD-associated synaptic plasticity dysfunction, ASD-like behaviors, and global translational control. We also utilized mice lacking one copy of *Gigyf2*, *Eif4e2* or co-deletion of one copy of each gene to further investigate ASD-like behaviors.

**Results:**

4EHP is expressed in excitatory neurons and synaptosomes, and its amount increases during development. 4EHP-eKO mice display exaggerated mGluR-LTD, a phenotype frequently observed in mouse models of ASD. Consistent with synaptic plasticity dysfunction, the mice displayed social behavior impairments without being confounded by deficits in olfaction, anxiety, locomotion, or motor ability. Repetitive behaviors and vocal communication were not affected by loss of 4EHP in excitatory neurons. Heterozygous deletion of either *Gigyf2*, *Eif4e2*, or both genes in mice did not result in ASD-like behaviors (i.e. decreases in social behavior or increases in marble burying). Interestingly, exaggerated mGluR-LTD and impaired social behaviors were not attributed to changes in hippocampal global protein synthesis, which suggests that 4EHP and GIGYF2 regulate the translation of specific mRNAs to mediate these effects.

**Limitations:**

This study did not identify which genes are translationally regulated by 4EHP and GIGYF2. Identification of mistranslated genes in 4EHP-eKO mice might provide a mechanistic explanation for the observed impairment in social behavior and exaggerated LTD. Future experiments employing affinity purification of translating ribosomes and mRNA sequencing in 4EHP-eKO mice will address this relevant issue.

**Conclusions:**

Together these results demonstrate an important role of 4EHP in regulating hippocampal plasticity and ASD-associated social behaviors, consistent with the link between mutations in *GIGYF2* and ASD.

## Background

Autism spectrum disorder (ASD) is a neurodevelopmental condition affecting 1–2% of the global population [1]. The fifth edition of the Diagnostic and Statistical Manual of Mental Disorders (DSM-5) defines ASD based on deficits in social interaction (including nonverbal social communication) and restrictive or repetitive patterns of behavior. Current medical practice relies primarily on behavioral assessment to diagnose ASD, and pharmaceutical treatment is often inadequate and does not target the underlying pathophysiology of the core deficits. This places precedence on the discovery of reliable biomarkers and more individualized medical interventions. In the case of idiopathic ASD, hundreds of gene mutations serve as potential biomarkers, but direct causal evidence is lacking. Understanding how these individual gene mutations contribute to ASD is paramount to the development of personalized medication.

The disruption of protein synthesis (mRNA translation or translation) in the brain by genetic perturbations of its regulators constitutes a known underlying etiology for ASD [2, 3]. For most mRNAs, initiation of translation requires binding of initiation factors to their 5′ end at a modified guanine nucleotide (m^7^GpppN, where N is any nucleotide) termed the 5′ cap [4]. The eukaryotic initiation factor (eIF) 4F complex is comprised of the cap binding protein eIF4E, an mRNA helicase eIF4A, and a molecular scaffold eIF4G. Together these proteins facilitate recruitment of the ribosomal 43S preinitiation complex to the mRNA. Overactivity of eIF4E in humans has been implicated in ASD [5, 6] and ASD-like phenotypes in mice [7, 8]. Indeed, disruption of the proteins regulating eIF4E activity, such as fragile X mental retardation protein (FMRP) [9], cytoplasmic FMR1 interacting protein 1 (CYFIP1) [10], and eIF4E-binding protein 2 (4E-BP2) [8, 11, 12], is implicated in ASD. It is therefore necessary to investigate the function of ASD-linked genes that encode for regulators of translation. Whole-genome sequencing of ASD patients has been invaluable in identifying these genes.

By inspecting these datasets, we identified 22 unique mutations in the gene encoding GRB10 interacting GYF protein 2 (GIGYF2) which have been associated with ASD [13–19]. The nature of these mutations is gene disruptive, such as large deletions, premature termination, and loss of termination-codon mutations. Although its mechanism of action is not fully understood, GIGYF2 forms a complex with the eIF4E homologous protein (4EHP) which is required for the stable expression of both proteins (i.e. deletion of one results in reduced expression of the other) [20]. 4EHP, encoded by the gene *Eif4e2* in mice, binds to the mRNA 5′ cap. Unlike eIF4E, 4EHP acts to repress translation [20] because it cannot recruit the scaffolding protein, eIF4G [21]. Instead, 4EHP requires interaction with GIGYF2 to repress translation of target mRNAs [22]. Therefore, loss of either GIGYF2 or 4EHP results in increased rates of protein synthesis [20, 23]. We hypothesized that *GIGYF2* mutations disrupt the coordinated function of the 4EHP and GIGYF2 protein complex, resulting in impaired synaptic function and susceptibility to ASD.

Here we investigated ASD-like phenotypes in various mutant mouse models for *Gigyf2* and *Eif4e2*. Our findings provide documentation of 4EHP expression in the brain and reveal an important role of 4EHP in excitatory neurons, namely in the regulation of synaptic plasticity and ASD-associated social behaviors. Together these findings are consistent with the genetic link between *GIGYF2* and ASD.

## Methods

### Mice

Male mice on Jackson Laboratory C57BL/6J background aged postnatal day (P) 60–90 (i.e. young adults [24]) were used for experiments, unless otherwise specified. *Gigyf2*^+/−^ [25] and *Eif4e2*^+/−^ [20] were previously generated and characterized. Mice were weaned at P21 and housed by sex and mixed genotype (unless otherwise specified) in groups of 2–5 animals per cage under standard conditions: 20–22 °C, 12 h light/dark cycle (7:00–19:00 light period) with food and water access ad libitum. Mice were handled 3 times (once per day for 3 days) and habituated to the behavioral room for 20 min prior to behavioral testing. Behavioral experiments were conducted in an isolated, soundproof room between 9:00 and 16:00. All behavioral apparatuses were cleaned between animals. In the case where cohorts were evaluated in more than one behavioral assay, the testing order began with the least aversive test and ended in the most aversive (least – grooming, open field, elevated plus maze, marble burying, rotarod, three-chamber social interaction, and contextual fear conditioning—most). All other behavioral tests were conducted on separate cohorts aged P60–P90, unless otherwise specified. See below for detailed methods. The experimenter was blinded to mouse genotype during data acquisition, analysis and manual scoring. Mouse genotype was randomized throughout the day and across days in the case of multi-day experiments. Animal care, handling, and all experiments were performed according to the guidelines of the Canadian Council on Animal Care and approved by the McGill University Animal Care Committee.

### Generating *Eif4e2* conditional knockout (KO) mice

To conditionally delete 4EHP in excitatory neurons, we crossed *Eif4e2*^*flx/flx*^ mice [20] with Emx1-IRES-Cre mice (glutamatergic forebrain neurons where Cre recombinase activity occurs at embryonic day (e) 10.5 [26], JAX stock no. 005628, on C57BL/6 background, backcrossed for 12 generations). *Eif4e2*^+*/flx*^*:Emx1-Cre* mice were used to breed F2: *Eif4e2*^+*/*+^*:Emx1-Cre* (referred to in the text as 4EHP-WT) and *Eif4e2*^*flx/flx*^*:Emx1-Cre* (referred to in the text as 4EHP-eKO). F3 mice were used for experiments and housed according to genotype. Comparisons were made between these genotypes to normalize for any confounding effects generated by the presence of Cre recombinase alone.

### Synaptic protein extraction

The hippocampus from mice (wild-type male on Jackson Laboratory C57BL/6J background, n = 3) was dissected and homogenized in ice-cold Syn-PER Synaptic Protein Extraction Reagent (87,793, Thermo) containing 1 tablet EDTA-free protease inhibitor mixture (4,906,845,001, Roche), phosphatase inhibitor mixture 2 (P5726, Sigma) diluted 1:100, and phosphatase inhibitor mixture 3 (P0044, Sigma) diluted 1:100. Following the manufacturer’s protocol, the samples were centrifuged at 1200 g for 10 min at 4 °C and the supernatant was transferred to a new tube. A sample was taken for crude. The supernatant was then centrifuged at 15 000 g for 10 min at 4 °C, and the supernatant (cytosol) was removed from the synaptosome pellet. The synaptosome pellet was then resuspended in Syn-PER Synaptic Protein Extraction Reagent for analysis. Samples were stored at – 80 °C until used for Western blotting.

### Western blot

**Fig. 1:**
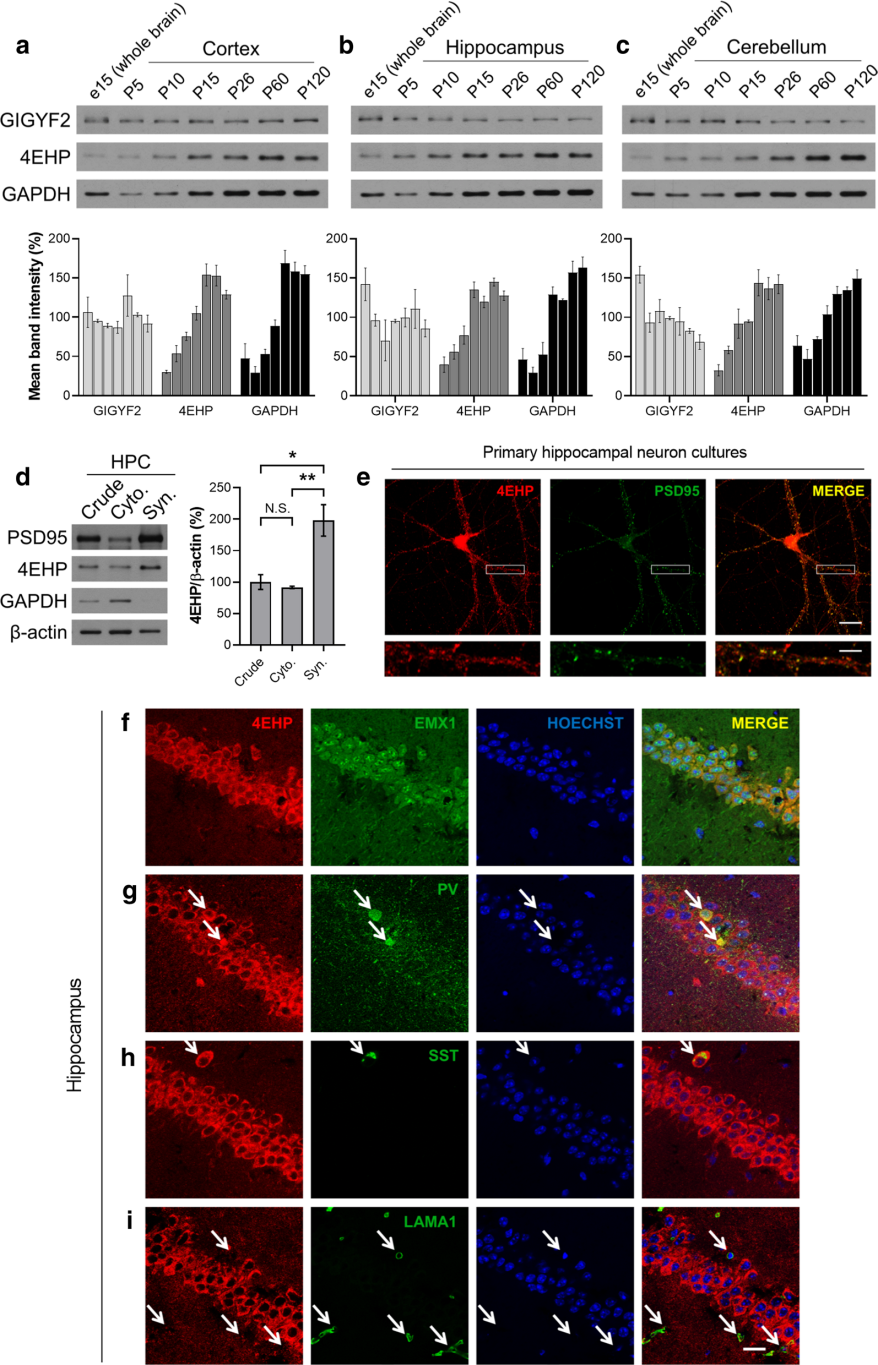
4EHP expression in the brain. **a**–**c** Developmental expression of 4EHP, GIGYF2, and GAPDH in the cortex, hippocampus, and cerebellum, respectively, as measured by western blot. Quantification of a, b, and c (lower panel, n = 3 per group, normalized to the average of all age points for each protein per membrane). **d** 4EHP expression in a synaptosome preparation (left panel). PSD95 was enriched in the synaptosome (Syn) as opposed to the cytosol (Cyto), demonstrating proper synaptosome preparation. GAPDH and β-actin were used as loading controls. Quantification of d (right panel, n = 3). **e** Primary neurons were derived from the hippocampus of wild-type mice and cultured for 14 d. Immunofluorescent analysis confirmed 4EHP expression in the synapse by colocalization with PSD95 (merge). The scale bar represents 20 µm in the upper panel of images and 5 µm in the lower panel of images. The lower panel of images correspond to 4 × zoom of the upper panel of images defined by the white box. Analysis of cell-type-specific expression of 4EHP by colocalization with **f** Empty Spiracles Homeobox 1 (EMX1, defining excitatory neurons), **g** parvalbumin (PV, defining a subset of inhibitory neurons), **h** somatostatin (SST, defining another subset of inhibitory neurons), and **I** laminin (LAMA1, defining endothelial cells) in the hippocampus of wild-type mice. 4EHP expression is colored in red, the cell type marker in green, and Hoechst-stained nucleus in blue. Arrows indicate a positive signal for the cell type maker. Scale bar represents 20 µm

Soluble protein extracts were prepared by homogenizing brain tissue (from 3 to 8 mice, depending on the experiment) using a pestle mixer in ice-cold radioimmunoprecipitation assay (RIPA) buffer (R0278, Sigma) containing proteinase and phosphatase inhibitors. Samples were incubated on ice for 30 min. Lysate was then centrifuged at 16 000 g for 20 min at 4 °C. The protein-containing supernatant was collected, and the pellet discarded. 25 µg of protein sample was loaded onto a polyacrylamide gel (final concentration: 12% Acrylamide/Bis Solution, 29:1, 375 mM Tris pH 8.8, 0.1% SDS, 0.1% TEMED, and 0.1% Ammonium Persulfate) and separated using a potential difference of 100 V. Protein was then electrotransferred onto a nitrocellulose membrane in transfer buffer (25 mM Tris, 190 mM glycine, and 20% methanol, pH 8.3) at 25 V overnight at 4 °C. Membranes were then blocked with 5% albumin (BSA) in Tris-Buffered Saline with Tween 20 (TBST, 20 mM Tris pH 7.5, 150 mM NaCl, 0.1% Tween 20) for 1–2 h at room temperature (RT) to reduce non-specific binding. Membranes were then probed with one of the following primary antibodies at the indicated dilution: EIF4E2 (GTX103977, GeneTex, 1:500), GIGYF2 (A303-732A, Bethyl Laboratories, 1:500), PSD95 (75-028, NeuroMab, 1:5000), α-Tubulin (sc-23948, Santa Cruz, 1:5000), GAPDH (ab9482, Abcam, 1:40 000), β-actin (A5441, Sigma, 1:5000), diluted in TBST with 5% BSA overnight at 4 °C (or 1 h at RT for GAPDH and β-actin), then washed with fresh TBST 3 times for 10 min each at RT. Secondary antibody conjugated to horseradish peroxidase (HRP, anti-mouse and anti-rabbit, GE Healthcare) was diluted 1:5000 in TBST with 5% BSA and added to membranes for 1–2 h at RT. Membranes were again washed with fresh TBST 3 times for 10 min each at RT. Enhanced chemiluminescence (Western Lighting® Plus ECL, 0RT2655:0RT2755, PerkinElmer) was then added to membranes for 1 min. Membranes were visualized on film. For re-probing, membranes were washed with double distilled water for 5 min, the antibody was stripped with 0.2 M NaOH for 10 min, and membranes washed again with double distilled water for 5 min. Quantification of the band intensity was done using Image J software (NIH). For analysis of developmental expression of GIGYF2 and 4EHP (Fig. 1a–c), wild-type male mice on Jackson Laboratory C57BL/6 J background were used at the indicated age (n = 3 per age group).

### Primary hippocampal neuron cultures

Hippocampi were dissected from wild-type e17.5 mouse brain on Jackson Laboratory C57BL/6J background in ice-cold Hank’s Balanced Salt Solution (HBSS). Hippocampi were washed in ice-cold HBSS without calcium and magnesium twice and cells were dissociated by incubating in trypsin at 37 °C. Trypsin digestion was stopped by adding fetal bovine serum (FBS). After washing twice with Dulbecco’s Modified Eagle Medium (DMEM) containing 10% FBS, the dissociated cells were plated on dishes pre-coated with polyethyleneimine overnight in DMEM containing 10% FBS. 2 h after plating, the media was removed and replaced by Neurobasal media containing B-27 supplement, GlutaMAX, and Penicillin/Streptomycin. After 2 d in vitro (DIV), cells were treated with mitotic inhibitor (5-Fluoro-2′-deoxyuridine) to prevent glial contamination. Half of the media was replaced with new media every 5 d until analysis.

### Immunofluorescence on primary neuron cultures

DIV 14 primary hippocampal neurons were briefly washed with preheated phosphate-buffered saline (PBS) at 37 °C. Cells were then fixed with preheated 4% paraformaldehyde (PFA) at 37 °C for 10 min. After washing with PBS 3 times for 10 min, cells were permeabilized with 0.2% triton X-100 in PBS at RT for 15 min. Cells were blocked in 1% BSA in PBS at RT for 1 h. Blocking buffer was then exchanged for the following primary antibodies: eIF4E2 (sc-100731, Santa Cruz), PSD95 (75-028, NeuroMab), diluted 1:100 in blocking buffer. Cells were incubated in primary antibody at 4 °C overnight. Cells were washed with PBS 3 times for 10 min before adding secondary antibody diluted 1:1000 in blocking buffer for 1 h at RT in the dark. Cells were washed with PBS 3 times for 10 min before being mounted on a microscope coverslip with DAKO. Cells were visualized using a ZEISS Laser Scanning Microscope 880 24 h after mounting.

### Immunofluorescence on brain slices

Mice were placed under isoflurane anesthetics until loss of pain reflex and transcardially perfused with filtered ice-cold PBS then 4% PFA. Brains were rapidly dissected and placed in ice-cold 4% PFA overnight at 4 °C for post-fixation. Brains were then placed in 30% sucrose in PBS for 3 d at 4 °C for cryoprotection. 20 µm coronal sections were prepared using a cryostat and adhered to glass coverslips (12-550-15, Fisher). Sections were washed 3 times in PBS for 5 min and placed in boiling 10 mM sodium citrate buffer, pH 6.0 for 20 min for antigen retrieval. Sections were washed 3 times with PBS for 5 min before placed in blocking solution (10% BSA and 0.5% Tween 20 in PBS) for 1–2 h at RT. Sections were then incubated in the following primary antibodies: eIF4E2 (sc-100731, Santa Cruz), EMX1 (PA5-35373, Thermo), PVALB (195004, Synaptic System), Somatostatin 28 (ab111912, Abcam), Laminin (L9393, Sigma), diluted 1:100 in blocking solution overnight at 4 °C. After washing 3 times in PBS for 5 min, sections were incubated with Alexa-conjugated secondary antibodies (1:300) and Hoechst (1:1000) diluted in blocking buffer for 1–2 h at RT in the dark. Sections were then washed 3 times with PBS for 5 min and then rinsed once in double distilled water. Coverslips were mounted with DAKO. Samples were visualized 24 h later with a ZEISS Laser Scanning Microscope 880.

### Electrophysiological recordings

Transverse hippocampal slices (400 µm thick) were prepared from age-matched male mice (4–5 weeks of age) with a vibratome (Leica VT1200 S, Leica Biosystems Inc) at 4 °C in artificial cerebrospinal fluid solution (ACSF, perfused with 95% O_2_ and 5% CO_2_) containing 124 mM NaCl, 5 mM KCl, 1.25 mM NaH_2_PO_4_·H_2_O, 2 mM MgSO_4_·7H_2_O, 26 mM NaHCO_3_, 2 mM CaCl_2_·H_2_O, and 10 mM Dextrose. Slices were recovered for at least 120 min before recording in an incubation chamber with ACSF at 32 °C. The slices were then transferred to the recording chamber and perfused with ACSF at a flow rate of 2 mL/min for 30 min prior to recording. Field excitatory postsynaptic potentials (fEPSPs) were recorded with ACSF-filled micropipettes and were elicited by bipolar stimulating electrodes placed in the CA1 stratum radiatum to excite the Schaffer collateral. Input–output curves were generated by increasing input current and recording fEPSP output. The intensity of the pulses was adjusted to evoke 40–50% of maximal response for subsequent recording. A stable baseline of responses was established for 30 min and metabotropic glutamate receptor-meditated long-term depression (mGluR-LTD) was induced by bath-application of 100 µM (*S*)-3,5-Dihydroxyphenylglycine (DHPG, 0805, Tocris Biosciences) for 10 min. Each data point represents the slope of fEPSP calculated with Clampfit 11.0.3 software. All data are presented as mean ± s.e.m. and n refers to the number of mice (i.e. 1 recording from 1 slice from 1 mouse).

### Measurement of global protein synthesis

The puromycin incorporation assay, also known as surface sensing of translation (SUnSET) [27], was performed on adult (P60-P90) hippocampal slices as previously described [28]. Briefly, 400 µm transverse hippocampal slices were prepared as indicated for electrophysiology experiments. Slices were recovered for a minimum of 3 h in an incubation chamber with ACSF at 32 °C. Six slices were combined per animal and each n represents one animal. Puromycin Dihydrochloride (PUR333.10, BioShop) was added to the incubation chambers at a final concentration of 5 µg/mL. Slices were incubated in puromycin for 45 min and then either snap frozen and prepared for western blot or placed in 4% PFA in preparation for immunofluorescence. Puromycin incorporation was visualized using western blot or immunofluorescence with an anti-puromycin antibody, clone 12D10 (1:1000, MABE343, MilliporeSigma).

### Three-chamber social interaction

An arena partitioned into three chambers containing doors to allow entry into each chamber was used to assess social interaction and preference for social novelty. Test mice were placed in the middle of the empty three-chambered arena and habituated for 10 min. After habituation, an unfamiliar mouse (stranger 1, age-matched male, C57BL/6J, and approximately the same size as the test mouse) was placed into one of the two side chambers and enclosed in a small holding device which only permitted social interaction to be initiated by the test mouse. An identical empty holding device was placed in the opposite chamber. During this time, the doors to the side chambers were blocked to prevent the test mouse from entering the chambers. The doors were then opened, and the test mouse could explore for 10 min. After 10 min, the doors were again blocked and a new unfamiliar mouse (stranger 2, age-matched male, C57BL/6J, and approximately the same size as the test mouse) was placed in the previously empty holding device. The doors were opened again, and the test mouse freely explored for 10 min. The location of the holding device was counterbalanced between side chambers for different test mice to prevent chamber biases. Stranger 1 and 2 mice were from different home cages and counterbalanced for each side of the chamber. The time spent sniffing stranger 1, stranger 2 or the empty holding device was manually scored. Stranger mice were purchased from Charles River Laboratories (Sherbrooke, Canada).

### Marble burying

An open field arena (50 cm by 50 cm by 30 cm) was filled with fresh bedding (i.e. sawdust, approximately 5 cm deep). Twenty clean marbles were placed on the sawdust in a pre-arranged 5 by 4 grid. Mice were placed in the center of the field and allowed to bury the marbles for 20 min. After the test period, buried marbles (i.e. marbles that were at least 2/3 covered with sawdust) were counted manually.

### Direct social interaction

The test mouse was placed in a new, clean cage and allowed to habituate for 5 min. A novel stranger mouse (age-matched male, C57BL/6J, and approximately the same size as the test mouse) was then placed in the cage and the mice interacted for 10 min. Activity and interaction was recorded using a camera placed vertically in front of the cage. Videos were scored manually to obtain the nose-to-anogenital sniffing time of the stranger mouse by the test mouse and total interaction time, including nose-to-nose sniffing, nose-to-anogenital sniffing, following, chasing, mounting, and fighting during the 10-min interaction. Reciprocal interaction of the stranger mouse to test mouse was also included in the total interaction time.

### Self-grooming

Clean home cages were filled with approximately 1 cm of fresh bedding material without nesting material. Mice were individually placed in a cage and recorded for 20 min using a video camera placed in front of the cage. Total grooming time was manually scored using a stopwatch.

### Isolation-induced ultrasonic vocalizations

To induce USVs, mouse pups (P7) were gently separated from their mothers for 15 min (kept on a heating pad). Pups were then placed individually in an anechoic styrofoam chamber (recording chamber) containing a microphone (Avisoft Bioacoustics CM16/CMPA) fixed inside the top. The microphone was connected to an ultrasound recording interface (Avisoft Bioacoustics UltraSoundGate 116Hb) which detects USVs emitted by mouse pups and recorded using a digital recording system (Avisoft Bioacoustics RECORDER). USVs were recorded for 5 min. Recordings were analyzed manually using the Avisoft Bioacoustics SASLab Pro software. The number of calls per min and average call duration were analyzed.

### Open field

Mice were placed in a white-colored square box (50 cm × 50 cm × 30 cm) with an open top and allowed to explore freely for 10 min while their locomotor activity was recorded with a camera placed directly above the field. The center zone is defined as a square measuring 30 cm × 30 cm that is in the middle of the arena. Time spent in the center of the field, total distance travelled, and number of entries into the center were scored using Noldus EthoVision XT software.

### Rotarod

Mice were first trained to walk on a 1¼ diameter rotating rod (Rotarod, IITC Life Science Inc, USA) with a constant rotation of 5 revolutions per min (rpm). The training period lasted for 3 min and mice that fell off were placed back on during this time. 1 h after training, mice were placed on the rod which began rotating at 5 rpm and accelerated by 0.2 rpm per sec to a maximum speed of 40 rpm until either the mice fell off or 5 min passed. The latency to fall was recorded as a measure of motor function.

### Olfactory preference

To test for intact olfaction in mice, either cinnamon extract (clear in color) or water was placed on a 2 cm by 2 cm patch of filter paper in a clean home cage. Mice were then placed in the cage for 5 min and observed for time spent sniffing the filter paper containing either cinnamon extract or water. Since novelty of the filter paper alone promotes sniffing and may mitigate any differences in time spent sniffing either water or an attractive odor, an aversive odor was chosen for this test.

### Elevated plus maze

The testing apparatus consists of two black open arms and two black enclosed, protected arms that are both approximately 0.6 m above the floor, meeting at a center zone to form a plus shape. The open arms had open edges. The testing room was lit with 1200 lx. The total time spent in the open and closed arms was scored manually. A transition to another arm was defined as all four limbs entering either an open or a closed arm.

### Contextual fear conditioning

Mice were placed in a sound-proof box containing an enclosed isolation chamber with an electric grid floor and overhead camera. Mice were recorded for 2 min before receiving a mild foot shock (0.7 mA, 1 s). After 1 min, mice were removed and placed back in their home cage. After 24 h, mice were placed back in the enclosure (context) and recorded for 4 min. The average percent freezing over 4 min was used as an assessment of long-term memory.

### Statistical analysis

Statistical analysis was performed on GraphPad Prism 8. An unpaired t-test was used to compare one experimental parameter. Mixed design two-way ANOVA was used to compare two experimental parameters (i.e. genotype as an independent variable and arms in the elevated plus maze test as a repeated measure). Bonferroni test was used for pair-wise post hoc analysis where there was a significant interaction in the data. A Welch’s corrected t-test was used where the difference in variance between groups was significantly different according to the Levene’s test. Data were expressed as mean ± s.e.m. and p values < 0.05 were considered statistically significant. Details of all statistics used are listed in Additional file 1: Table 1.

**Table 1:** Mutations in *GIGYF2* are linked to autism spectrum disorder

*GIGYF2* chromosome location	Genomic mutation	Amino acid change	Mutation type	Inheritance pattern	References
2:233612356	A to G	Thr25Ala	Missense	Maternal	Wang et al. 2016, *Nat Commun*
2:233612356	A to G	Thr25Ala	Missense	Paternal	Wang et al. 2016, *Nat Commun*
2:233612456	T to C	None	Splice donor	N.D	Wang et al. 2016, *Nat Commun*
2:233613755	C to T	Pro77Leu	Missense	Maternal	Wang et al. 2016, *Nat Commun*
2:233651280—233673273	Deletion	Unknown	Intron deletion	De novo	Gazzellone et al. 2014, *J Neurodev Disord*
2:233655527	A to G	Ile300Val	Missense	Maternal	Wang et al. 2016, *Nat Commun*
2:233655527	A to G	Ile300Val	Missense	Paternal	Wang et al. 2016, *Nat Commun*
2:233655615	T to G	Leu329Arg	Missense	Maternal	Wang et al. 2016, *Nat Commun*
2:233655745	G to T	Glu320Ter	Nonsense	De novo	Wang et al. 2016, *Nat Commun*
2:233656136	A to G	Lys442Arg	Missense	Maternal	Wang et al. 2016, *Nat Commun*
2:233656136	A to G	Lys442Arg	Missense	Paternal	Wang et al. 2016, *Nat Commun*
2:233659563	G to A	Arg484Gln	Missense	Paternal	Wang et al. 2016, *Nat Commun*
2:233671257	T to G	Ser587Ala	Missense	Maternal	Wang et al. 2016, *Nat Commun*
2:233671353	C to T	Pro619Ser	Missense	Maternal	Wang et al. 2016, *Nat Commun*
2:233675964	G to A	Ala658Thr	Missense	De novo	Wang et al. 2016, *Nat Commun*
2:233675982	C to T	Gln664Ter	Nonsense	De novo	Iossifov et al. 2014, *Nature*
					Lim et al. 2017, *Nat Neurosci*
					An et al. 2018, *Science*
2:233677147	G to A	Val706Ile	Missense	Paternal	Wang et al. 2016, *Nat Commun*
2:233684582	C to T	Arg827Ter	Nonsense	De novo	Lim et al. 2017, *Nat Neurosci*
2:233704609	G to C	Gln960His	Missense	Maternal	Wang et al. 2016, *Nat Commun*
2:233704659	G to A	Arg977Gln	Missense	De novo	De Rubeis et al. 2014, *Nature*
					Wang et al. 2016, *Nat Commun*
					Lim et al. 2017, *Nat Neurosci*
2:233709081 – 233709092	Deletion	Ser1035—His1038	Exon deletion	N.D	Wang et al. 2016, *Nat Commun*
2:233712060	C to A	Pro1176Thr	Missense	Maternal	Wang et al. 2016, *Nat Commun*
2:233712,061	C to G	Pro1176Arg	Missense	De novo	Krumm et al. 2015, *Nat Genet*
					An et al. 2018, *Science*
2:233721568	T to G	Ter to Gly	Stop lost	N.D	Wang et al. 2016, *Nat Commun*
2:233721568	T to A	Ter to Arg	Stop lost	N.D	Wang et al. 2016, *Nat Commun*

NCBI Gene Assembly GRCh37.p13

N.D not determined

## Results

### 4EHP is primarily expressed in neurons and synaptosomes and its amount increases during development

To study the effects of homozygous deletion of 4EHP in the brain, we employed Cre-Lox technology. We first investigated the expression of 4EHP to provide a basis for generating an appropriate model. In the cortex (Fig. 1a), hippocampus (Fig. 1b), and cerebellum (Fig. 1c), 4EHP expression increases through development. Interestingly, 4EHP is maximally expressed between P26 and P60. In the hippocampus, 4EHP protein expression is enriched in purified synaptosomes (Fig. 1d), but is also expressed in the cytosol, consistent with previous reports [29]. We confirmed synaptic expression of 4EHP in primary hippocampal neuron cultures by colocalization of the synaptic marker PSD95 (Fig. 1e). Lastly, we examined 4EHP expression in major cell types in the hippocampus. 4EHP was observed primarily in neurons, including excitatory neurons, labelled by empty spiracles homeobox 1 (EMX1, Fig. 1f), and inhibitory neurons, labelled by either parvalbumin (PV, Fig. 1g) or somatostatin (SST, Fig. 1h). We did not observe 4EHP in a non-neuron cell type, endothelial cells, labelled by laminin (LAMA1, Fig. 1i). Given these results, we opted to target 4EHP in EMX1-expressing cells to study its role in synaptic plasticity and ASD-like behaviors. We chose the EMX1-Cre model over the CaMKIIa-Cre model to delete 4EHP in excitatory neurons because EMX1-driven Cre recombinase activity was reported to occur by e10.5 [26], whereas CaMKIIa-driven Cre recombinase activity occurs postnatally [12, 30, 31].

### 4EHP in excitatory neurons regulates hippocampal mGluR-LTD and is necessary for normal social behaviors

**Fig. 2:**
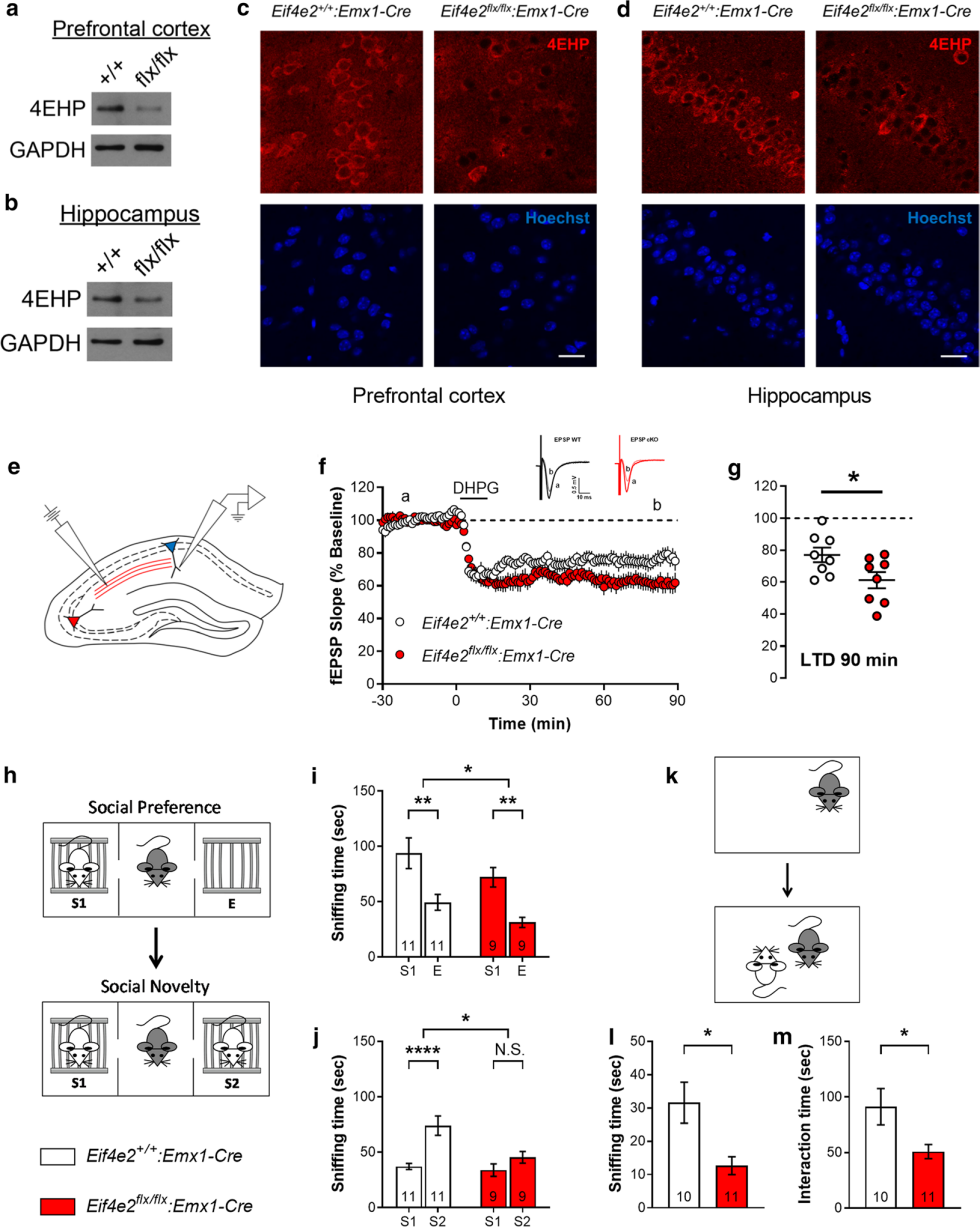
Loss of 4EHP in excitatory neurons exaggerates hippocampal mGluR-LTD and impairs social behavior. **a**, **b** Confirmation of loss of 4EHP expression in the prefrontal cortex and hippocampus, respectively, of 4EHP-eKO (flx/flx) versus 4EHP-WT (+ / +) mice using western blot. GAPDH was used as a loading control. **c**, **d** Confirmation of loss of 4EHP expression in excitatory neurons in the prefrontal cortex and hippocampus, respectively, of 4EHP-eKO versus 4EHP-WT mice using immunofluorescence microscopy. 4EHP expression is colored in red and Hoechst-stained nucleus in blue. Scale bar represents 20 µm. **e** Schematic representation of stimulating (left) and recording (right) electrode position for measuring DHPG-induced long-term depression (mGluR-LTD) in the CA1 hippocampus. The red fibers represent CA3 pyramidal projections to the CA1 (Schaffer collaterals). **f** Field excitatory postsynaptic potential (fEPSP) recordings of CA1 pyramidal neurons during mGluR-LTD. Baseline was recorded for 30 min prior to adding mGluR1/5 agonist, DHPG (100 µM), to slices for 10 min. LTD was recorded for 90 min. The inset is the average of all fEPSPs at time *a* and *b* for each genotype, n = 8 per group. **g** Average of the last 10 min of recording. **h** Schematic representation of the three-chamber social preference and social novelty test. Mice were first habituated to the apparatus for 10 min. Two cages (mouse holding devices) were then placed in opposite corners of opposing chambers; one cage was empty (E) and one contained a conspecific stranger mouse (S1). After 10 min, a novel stranger mouse (S2) was added to E for the social novelty test lasting 10 min. **i** The amount of time the test mouse spent sniffing either S1 or E. **j** The amount of time the test mouse spent sniffing either S1 or S2. **k** Schematic representation of the direct (reciprocal) social interaction test. Test mice were first habituated to a clean home cage for 5 min. A novel stranger mouse was then added, and mice could freely interact for 10 min. **l** Nose-to-anogenital sniffing time of the stranger mouse by the test mouse. **m** Total interaction time including nose-to-nose sniffing, nose-to-anogenital sniffing, following, chasing, mounting, and fighting. Reciprocal interaction of the stranger mouse to test mouse was also included. Data are presented as mean ± s.e.m.; *p < 0.05, **p < 0.01, ****p < 0.0001, N.S., not significant; calculated by unpaired t-test or 2-way ANOVA with Bonferroni multiple comparisons test. Sample size is located within bar graphs. *Eif4e2* is the mouse gene encoding 4EHP

The generation and characterization of mice expressing Cre in EMX1-specific cell types was previously reported [26]. By crossing these mice with those expressing a floxed *Eif4e2* (*Eif4e2*^*flx/flx*^), we generated an excitatory neuron-specific 4EHP knockout (4EHP-eKO) mouse model. Western blot analysis confirmed reduction of 4EHP expression in both the prefrontal cortex (Fig. 2a) and hippocampus (Fig. 2b). Loss of 4EHP expression in excitatory neurons was confirmed using immunofluorescence in both the prefrontal cortex (Fig. 2c) and hippocampus (Fig. 2d). We confirmed a reduction of both 4EHP and GIGYF2 expression in whole brain of P0 mice (Additional file 2: Fig. 1 A, B and C) and the hippocampus of P60 4EHP-eKO mice (Additional file 2: Fig. 1 D, E and F).

Given the hippocampal expression characteristics of 4EHP, we first investigated its role in hippocampal plasticity. Long-term depression (LTD) is a plasticity phenomenon that is exaggerated in mouse models of ASD with alterations in translational control [32, 33]. Depression of hippocampal neuron activity is also known to be necessary for normal social behavior in freely-moving rats [34] and is exaggerated in rats raised in social isolation [35]. To measure LTD, we recorded fEPSPs from CA1 pyramidal neurons after stimulating CA3 Schaffer collaterals (Fig. 2e). Application of 100 µM DHPG for 10 min resulted in a sustained reduction in the slope of fEPSPs (Fig. 2f). LTD was significantly exaggerated by 15.74% in 4EHP-eKO mice compared to 4EHP-WT (Fig. 2g). Given the correlation between normal hippocampal LTD and typical social behavior and the link between exaggerated mGluR pathway activation and ASD, we next investigated social behavior in 4EHP-eKO mice. To this end, we subjected mice to the three-chamber social preference and social novelty test (Fig. 2h). In the social preference phase, 4EHP-eKO preferred S1 over E, comparable to 4EHP-WT mice, but had 27.79% less overall interaction time with both S1 and E (Fig. 2i). However, in the social novelty phase, 4EHP-eKO mice did not exhibit a normal preference of the novel stranger mouse (S2) over S1 (Fig. 2j). Similarly, when allowed to freely interact with a stranger mouse in the direct or reciprocal social interaction test (Fig. 2k), 4EHP-eKO mice spent 59.91% less time sniffing and 44.22% less time interacting with the stranger mouse compared to 4EHP-WT mice (Fig. 2l, m). Together these results demonstrate an important role for 4EHP in mediating social behavior and regulating synaptic plasticity.

We next investigated global protein synthesis in 4EHP-eKO and 4EHP^+/−^ mice by measuring puromycin incorporation into nascent peptides of the hippocampus using the SUnSET assay [27]. We did not observe changes to global protein synthesis by western blot (Additional file 3: Fig. 2A) or immunofluorescence (Additional file 3: Fig. 2C) in 4EHP-eKO mice or 4EHP^+/−^ mice (Additional file 3: Fig. 2B) compared to controls. These findings suggest that 4EHP likely represses translation of specific mRNAs rather than global protein synthesis in the brain.

### ASD-like behavioral impairments in 4EHP-eKO mice are specific to social interaction and are not confounded by deficits in locomotion, motor function, olfaction, or anxiety

**Fig. 3:**
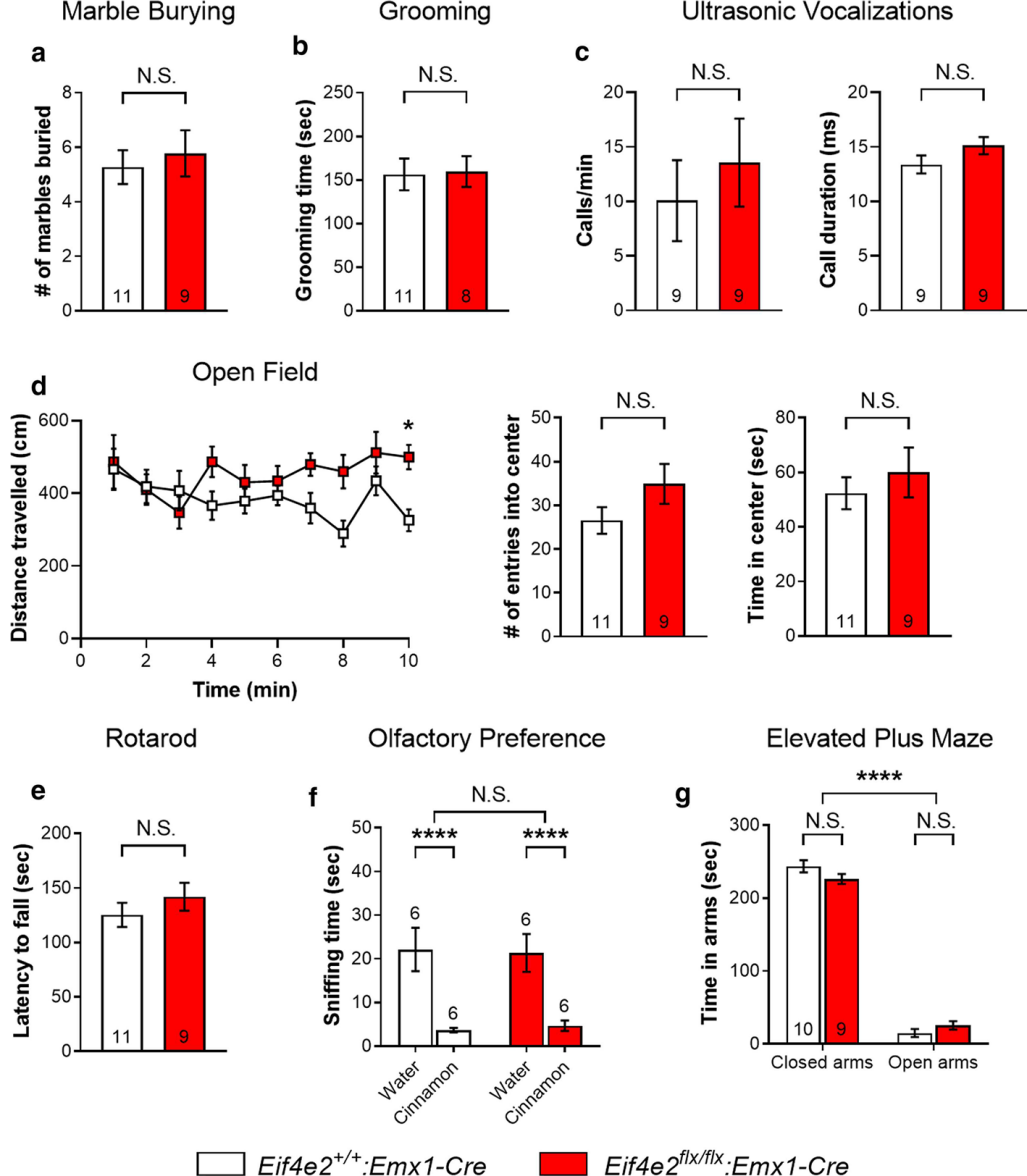
4EHP-eKO mice do not present wide-spread behavioral alterations. **a** To investigate repetitive behaviors, mice were analyzed in the marble burying assay. Mice were placed in an open field containing approximately 3 cm of fresh bedding material with 20 marbles in an evenly spaced 4 by 5 grid on the surface. Mice could bury marbles for 20 min. **b** Mice were placed in a clean home cage and total time spent grooming was recorded for 20 min. **c** P7 mice were separated from their mother and habituated for 15 min to induce vocalizations. The calls per min (left panel) and call duration (right panel) were recorded for 5 min. **d** Mice were placed into an open field for 10 min to assess locomotion and generalized anxiety. The distance travelled over time (left panel), number of entries into the center of the field (middle panel), and cumulative time spent in the center (right panel) were recorded; *p < 0.05, between groups at t = 10 min, calculated by 2-way ANOVA with Bonferroni multiple comparisons test. **e** Mice were placed on a rod rotating at a constant speed for 5 min for habituation. Mice were then placed back on the rod of increasing rotation speed until mice fell. The latency to fall was recorded as a measure of motor function. **f** To test olfaction, mice were placed into a clean home cage containing a piece of filter paper with a drop of either water or pure cinnamon extract. Time spent sniffing the filter paper was recorded for 5 min. **g** Generalized anxiety was assessed in the elevated plus maze by comparing time spent in an open versus closed arm for 5 min. Data are presented as mean ± s.e.m.; **p < 0.01, ****p < 0.0001, N.S., not significant; calculated by unpaired t-test or 2-way ANOVA with Bonferroni multiple comparisons test. Sample size is located within or above bar graphs

To further assess ASD-like behaviors in 4EHP-eKO mice, we investigated repetitive behaviors (marble burying and grooming) and ultrasonic vocalizations. 4EHP-eKO mice buried the same number of marbles (Fig. 3a) and self-groomed for the same duration (Fig. 3b) as 4EHP-WT mice. Ultrasonic vocalizations (USVs) were not different between 4EHP-eKO and 4EHP-WT mice (calls/min, Fig. 3c left panel; call duration, Fig. 3c right panel). As a measure of locomotion, distance travelled was not different between groups in an open field except during the last min of exploration where 4EHP-eKO mice travelled significantly further than 4EHP-WT mice (Fig. 3d left panel, P = 0.0128). As a measure of gross motor function, the latency to fall off a rotating rod of increasing speed was also not different between groups (Fig. 3e). Olfaction was not different between groups (Fig. 3f) as determined by the difference in time spent sniffing a neutral scent (water) and a repulsive scent (cinnamon extract). The elevated plus maze and open field were used to assess general anxiety as anxious mice spend less time in the open arms or less time in the center of an open field, respectively [36, 37]. We did not observe general anxiety in the 4EHP-eKO mice compared to 4EHP-WT in either the elevated plus maze (Fig. 3g) or in the open field (Fig. 3d middle and right panel). Since 4EHP was previously shown to regulate p-ERK [23], we measured hippocampal-dependent contextual fear memory in 4EHP-eKO mice, which requires activation of ERK [38–40]. Percent freezing 24 h after receiving an adverse stimulus (foot shock) was not different between 4EHP-eKO and 4EHP-WT mice (Additional file 4: Fig. 3A). Consistently, we did not observe a significant difference in p-ERK levels in the hippocampus of 4EHP-eKO mice (Additional file 4: Fig. 3 B-E).

### *GIGYF2* mutations are linked to ASD, but heterozygous deletion of *Gigyf2*, *Eif4e2*, or both in mice does not elicit ASD-like behaviors

**Fig. 4:**
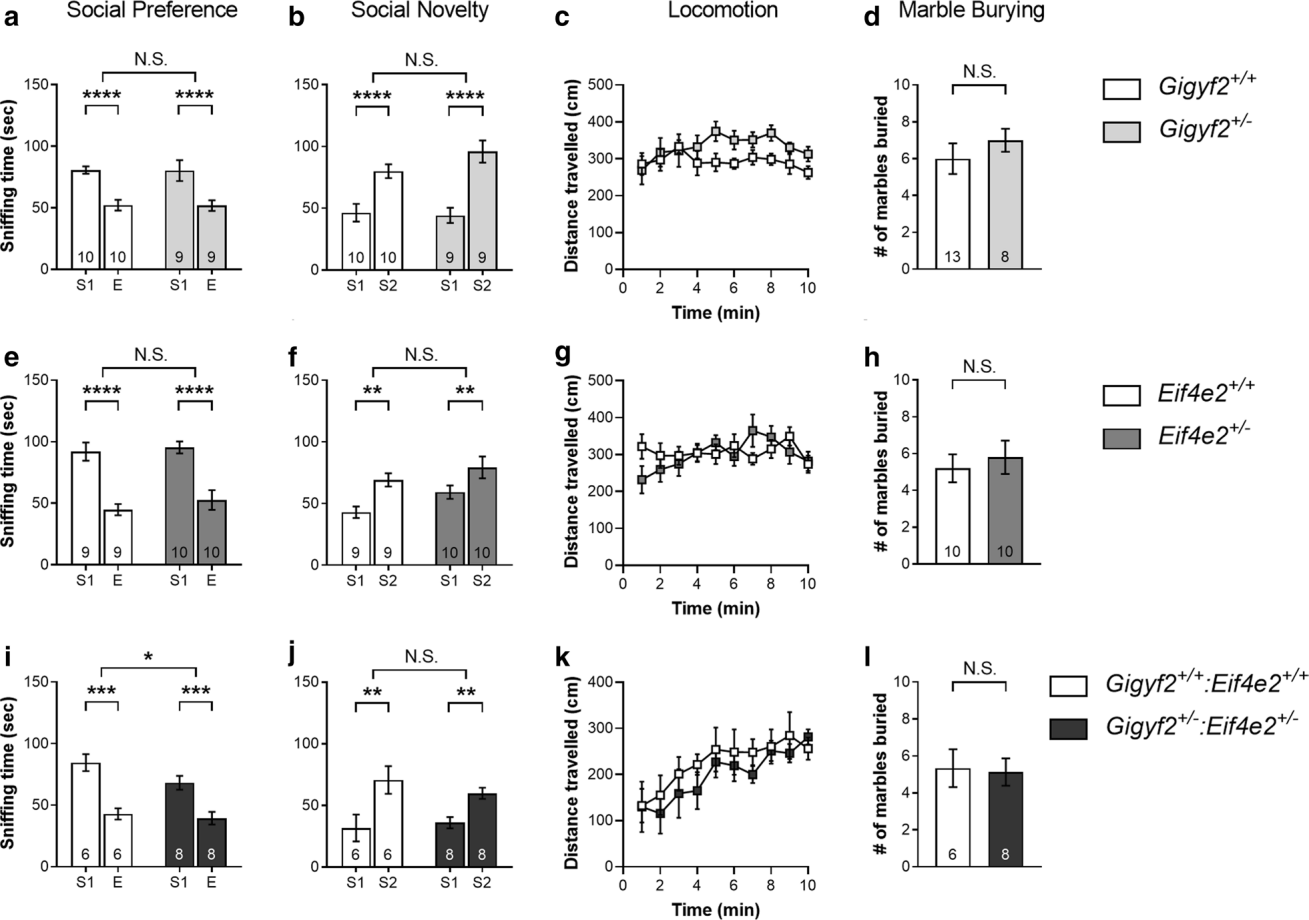
Heterozygous deletion of *Gigyf2*, *Eif4e2*, or both in mice does not result in ASD-like behavioral deficits. **a**, **e** and **i** The amount of time the test mouse of the specified genotype spent sniffing either S1 or E. **b**, **f**, **j** The amount of time the test mouse of the specified genotype spent sniffing either S1 or S2. **d, h**, **l** The number of marbles buried by the specified genotypes in 20 min. **c**, **g**, **k** Distance travelled over time during the 10 min habituation phase of the three-chamber social interaction test by the specified genotypes. Data are presented as mean ± s.e.m.; *p < 0.05, **p < 0.01, ***p < 0.001, ****p < 0.0001, *NS* not significant; calculated by unpaired t-test or 2-way ANOVA with Bonferroni multiple comparisons test. Sample size is located within bar graphs

**Fig. 5:**
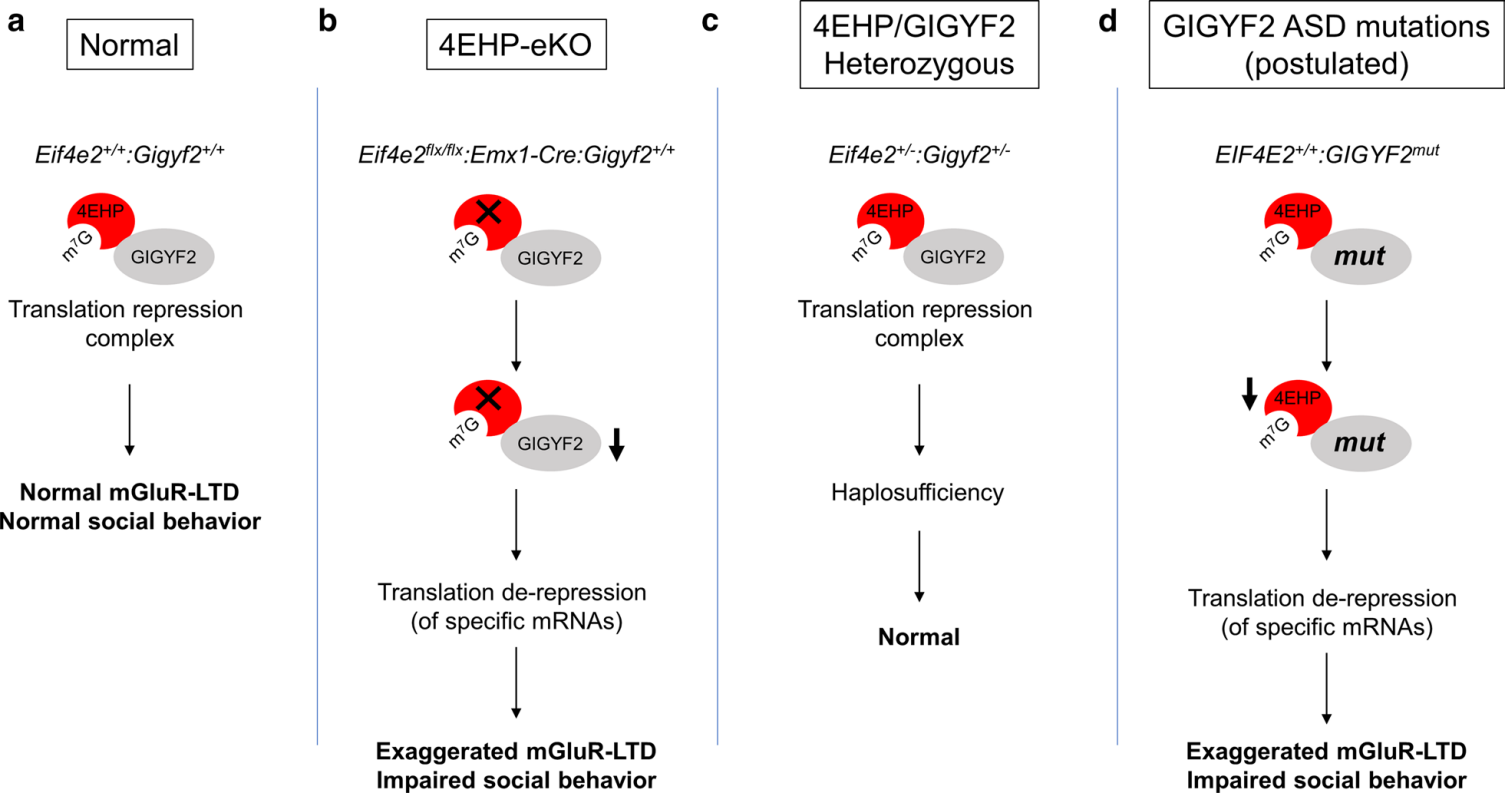
Proposed model. **a** 4EHP binds to the mRNA 5′ cap where its stable expression and function is maintained and reciprocated by physical interaction with GIGYF2. **b** Homozygous deletion of 4EHP in excitatory neurons of the forebrain (4EHP-eKO) results in reduced protein expression of GIGYF2, exaggerated mGluR-LTD, and impaired social behavior (possibly due to translation de-repression of specific mRNAs without affecting global protein synthesis). **c** Heterozygous deletion of *Gigyf2*, *Eif4e2*, or both does not result in ASD-like behaviors (possibly due to haplosufficiency). **d** Proposed model for the development of ASD in patients harboring *GIGYF2* mutations

Formation of a complex between 4EHP and GIGYF2 is required for the stability of both proteins [20] (Additional file 2: Fig. 1). As a translational repressing mechanism, disruption of this complex is a potential underlying cause of ASD (Fig. 5d). Various mutations in *GIGYF2* have been observed in ASD patients including truncations, large deletions, alternative splice donors, and loss of a stop codon (Table 1), with each having a potentially deleterious effect on GIGYF2 expression and function. To test whether loss of *Gigyf2* results in ASD-like behaviors in mice, we investigated social and repetitive behaviors in *Gigyf2*^+/−^ compared to *Gigyf2*^+*/*+^, since homozygous deletion of *Gigyf2* is lethal [25]. We did not observe either impaired social interaction in the three-chamber social preference and social novelty test (Fig. 4a, b) or exaggerated repetitive behaviors in the marble burying test (Fig. 4d). Similar to *Gigyf2* KO, homozygous deletion of *Eif4e2* is lethal in mice [20]. To determine whether loss of 4EHP alone or in concert with GIGYF2 results in ASD-like behaviors, we assessed social and repetitive behaviors in *Eif4e2*^+/−^ compared to *Eif4e2*^+*/*+^ mice and *Gigyf2*^+/−^*:Eif4e2*^+/−^ compared to *Gigyf2*^+*/*+^*:Eif4e2*^+*/*+^ mice. Consistent with findings in *Gigyf2*^+/−^ mice, heterozygous deletion of *Eif4e2* did not result in abnormal social preference (Fig. 4e), preference for social novelty (Fig. 4f) or increased marble burying (Fig. 4h). Heterozygous deletion of both *Gigyf2* and *Eif4e2* also did not result in impaired social behavior (Fig. 4i, j), although *Gigyf2*^+/−^*:Eif4e2*^+/−^ spent less time overall interacting with both stranger 1 (S1) and the empty cage (E) (Fig. 4i). The mice also did not present with differences in the number of marbles buried (Fig. 4l). As a measure of locomotion, distance travelled during the habituation phase of the three-chamber social interaction test was not different between groups (Fig. 4c, g, k). Together these results indicate that heterozygous deletion of *Gigyf2*, *Eif4e2*, or both is not sufficient to cause ASD-like behaviors in mice (Fig. 5c).

## Discussion

The behavioral deficits observed in 4EHP-eKO mice were specific to sociability, and not confounded by alterations in other behavioral domains. Impairments in either locomotion or motor activity may confound social interaction, since the mice are required to explore unhindered. The social behavior tests utilized here rely on intact olfaction as time spent sniffing is the dependent variable. Likewise, compounds that reduce general anxiety, such as the GABA_A_ receptor allosteric modulator ganaxolone, are known to have a confounding effect on social behavior [41]. We therefore tested and controlled for each of these potential confounding variables using the open field (Fig. 3d), rotarod (Fig. 3e), olfactory preference test (Fig. 3f), and the elevated plus maze (Fig. 3g), respectively. We conclude that 4EHP-eKO mice have specific social deficits.

In fact, the only behavioral phenotype relevant for ASD observed in 4EHP-eKO was impaired sociability. Both marble burying and self-grooming, which are used to assess repetitive behaviors, were unaltered in these mice. Since these behaviors are highly dependent on midbrain structures, such as the basal ganglia [42–44], restricted deletion of 4EHP in the forebrain of the eKO model [26] is not expected to affect these behaviors. Another possibility is that 4EHP activity in other cell types, such as inhibitory neurons, is mediating these behaviors. This is the case for 4E-BP2 conditional KO mice where 4E-BP2 deletion in inhibitory neurons resulted in impaired USVs, but not when it is deleted in excitatory neurons [12]. Consistent with these findings, USVs were not affected in 4EHP-eKO mice (Fig. 3c).

We confirmed social behavior deficits in two similar but distinct sociability tests: the three-chamber social interaction test and the direct or reciprocal interaction test. In the three-chamber social interaction test, 4EHP-eKO mice were not impaired in the first phase, which tests the animal’s preference for social interaction over interaction with an inanimate object. However, in the second phase, which tests the animal’s preference for social novelty, 4EHP-eKO mice did not prefer to interact with a novel stranger mouse over the one previously encountered. This phenotype is also observed in FMRP KO mice [45]. The reduction in nose-to-anogenital sniffing in 4EHP-eKO is also consistent with findings in other models of ASD, including in *Shank3* KO mice [46].

Long-term contextual fear memory was not affected by deletion of *Eif4e2* in excitatory neurons (Additional file 4: Fig. 3a). This finding was unexpected because 4EHP is known to regulate the levels of phospho-extracellular-signal-regulated kinase (p-ERK) via translational upregulation of dual-specificity phosphatase (DUSP) 6 in mouse embryonic fibroblasts (MEFs) [23]. Similarly, siRNA knockdown of GIGYF2 in human embryonic kidney (HEK) 293 T cells decreased levels of p-ERK [47]. Since activation of ERK signaling is required for long-term memory [38–40], it is anticipated that loss of 4EHP in the hippocampus would result in long-term memory impairments. However, we did not observe changes to p-ERK levels in the hippocampus of 4EHP-eKO mice compared to controls (Additional file 4: Fig. 3B-E). It is possible that in neurons, the molecular mechanism of 4EHP is different than in MEFs or HEK293T cells. Another possibility is that 4EHP regulates long-term memory in inhibitory neurons, since previous findings demonstrated the importance of translational control in SST neurons for long-term memory [48].

4EHP-eKO mice displayed exaggerated hippocampal mGluR-LTD together with impaired social behavior (Fig. 2). Field potential recordings in the hippocampus of freely moving rats have demonstrated that during normal social behavior, hippocampal responses are inhibited [34]. Similarly, rats that were socially isolated from P2-9 had exaggerated LTD in amygdalo-hippocampal synapses while undergoing social behavior [35]. Together these findings suggest that depression of synaptic responses in the hippocampus is necessary for normal social behavior, but excessive inhibition occurs during impaired social development. These findings are consistent with the mGluR theory of Fragile X Syndrome (FXS) which suggests that exaggerated mGluR-LTD is a hallmark feature of ASD animal models with dysregulated translation control [33]. This theory has been supported by numerous studies in the FXS mouse model [28, 49–51] and other ASD mouse models where translational repressors are deleted, such as CYFIP1 [52] and 4E-BP2 [11]. We therefore conclude that 4EHP function in forebrain excitatory neurons is required for social behavior by regulating hippocampal long-term depression (Fig. 5b).

We did not observe changes in global protein synthesis in the hippocampus of either 4EHP-eKO or 4EHP^+/−^ mice. Since 4EHP^+/−^ do not have any behavioral impairments, these findings are not surprising and are likely due to haplosufficiency. In the 4EHP-eKO mice, these observations are consistent with a role for 4EHP in regulating the translation of specific mRNAs via micro RNA silencing [53]. Future experiments employing cell-type-specific ribosome profiling (such as viral Translating Ribosome Affinity Purification, vTRAP [54]) and mRNA sequencing will be required to address this hypothesis. It is also possible that 4EHP regulates local translation, as we observed its expression in synapses. In this case, changes to global protein synthesis may only be observed under stimulated conditions, such as upon activation of mGluRs, and would require more sensitive techniques than SUnSET.

There are currently no approved pharmaceutical treatments for the hallmark features of ASD and the available therapeutic options are limited to treating comorbidities. Together with its high prevalence rate, ASD poses a socio-economic burden across the globe. The complex genetic landscape of ASD creates further difficulty in effectively treating a heterogeneous population without reliable biomarkers. Understanding the pathophysiology of individual genetic aberrations is one step toward individualized medicine and more precise and targeted therapeutic interventions. This is reinforced by the unlikelihood of having a single treatment or therapy work for a variety of ASD patients [55, 56]. To this end, much work has identified prospective therapeutics for treating ASD and other neurological disorders, such as metformin [57, 58]. The data and models obtained from this work may provide a basis for preclinical pharmacogenetic studies to reverse ASD-like symptoms that could potentially benefit the health of individuals with ASD, particularly those harboring *GIGYF2* mutations (Fig. 5d).

## Limitations

In this study, we did not elucidate the molecular mechanism of 4EHP and GIGYF2 in the brain and how their dysregulation underlies the ASD-like phenotypes observed in 4EHP-eKO mice. To understand how 4EHP and GIGYF2 regulate ASD-like behaviors and LTD at the molecular level, future studies could employ viral Translating Ribosome Affinity Purification (vTRAP) to tag and capture mRNAs undergoing active translation [54]. This technique utilizes an adeno-associated virus (AAV) to express an eGFP-tagged ribosomal protein under the control of Cre recombinase. By purifying and sequencing ribosome-bound mRNAs, we can compare the translational efficiency (TE) of a gene across different treatment groups or genotypes [59]. This would allow for cell-type-specific and regionally selective gene expression analysis.

## Conclusions

Here we describe a novel mouse model featuring established phenotypes of ASD, such as exaggerated hippocampal mGluR-LTD and social behavior deficits. Taken together, our findings provide evidence to support a link between human mutations in *GIGYF2* and the development of ASD via dysregulation of the 4EHP/GIGYF2 complex (Fig. 5).

## Supplementary information

**Additional file 1**. Details of statistical analyses.

**Additional file 2**. **Figure 1**: Codeletion of 4EHP and GIGYF2 occurs as early as P0 in the brain of 4EHP-eKO mice. **A** Western blot analysis of GIGYF2 and 4EHP levels in P0 whole brain from 4EHP-WT (+/+) versus 4EHP-eKO (flx/flx) mice. GAPDH was used as loading control. **B**and **C** Quantification of band intensity from A, presented as percent control. **D** Western blot analysis of GIGYF2 and 4EHP levels in P60 hippocampus from 4EHP-WT (+/+) versus 4EHP-eKO (flx/flx) mice. GAPDH was used as loading control. **E** and **F** Quantification of band intensity from D, presented as percent control. Data are presented as mean ± s.e.m.; **p<0.01, ****p<0.0001; calculated by unpaired t-test. Sample size is located within bar graphs.

**Additional file 3**. **Figure 2**: Analysis of global protein synthesis.** A** Puromycin incorporation into hippocampal slices from 4EHP-WT and 4EHP-eKO mice measured by western blot (left panel) and quantification (right panel) normalized to GAPDH. **B** Puromycin incorporation into hippocampal slices from 4EHP^+/+^ and 4EHP^+/-^ mice measured by western blot (left panel) and quantification (right panel) normalized to GAPDH. **C** Puromycin incorporation into hippocampal slices from 4EHP-WT and 4EHP-eKO mice measured by immunofluorescence (left panel) and quantification (right panel). Puromycin staining is colored in red and Hoechst-stained nucleus in blue. Quantification of puromycin integrated density was performed on whole image using image J. Scale bar represents 20 µm. Data are presented as mean ± s.e.m.; N.S., not significant; calculated by unpaired t-test. Sample size is located within bar graphs.

**Additional file 4**. **Figure 3**: Analysis of long-term contextual fear memory and p-ERK. **A** Mice were placed into a soundproof box (context) with an electric grid floor. Freezing time was recorded for 2 min (Naïve) before receiving a mild foot shock (0.7 mA, 1 sec). Mice were placed back in the box after 24 hr and freezing behavior recorded, n=11 (4EHP-WT), n=9 (4EHP-eKO). **B** Western blot analysis of ERK activation (p-ERK) in the hippocampus of 4EHP-eKO versus 4EHP-WT mice. **C** Quantification of 4EHP normalized to GAPDH. **D** Quantification of p-ERK normalized to total ERK. **E** Quantification of total ERK normalized to GAPDH. Data are presented as mean ± s.e.m.; *p<0.05, N.S., not significant; calculated by unpaired t-test. Sample size is located within bar graphs.

## Data Availability

All data generated, collected, and analyzed here are available from the corresponding author upon reasonable request.

## Abbreviations

eIF4E2: Eukaryotic initiation factor 4E homologous protein
GIGYF2: GRB10 interacting GYF protein 2
ASD: Autism spectrum disorder
DSM-5: Diagnostic and statistical manual of mental disorders
FMRP: Fragile X mental retardation protein
CYFIP1: Cytoplasmic FMR1 interacting protein 1
4E-BP2: EIF4E-binding protein 2
P: Postnatal day
e: Embryonic day
KO: Knockout
RIPA: Radioimmunoprecipitation assay
BSA: Bovine serum albumin
TBST: Tris-buffered saline with Tween 20
RT: Room temperature
HRP: Horseradish peroxidase
HBSS: Hank’s balanced salt solution
FBS: Fetal bovine serum
DMEM: Dulbecco’s modified eagle medium
DIV: Days in vitro
PBS: Phosphate-buffered saline
PFA: Paraformaldehyde
ACSF: Artificial cerebrospinal fluid solution
fEPSPs: Field excitatory postsynaptic potentials
mGluR-LTD: Metabotropic glutamate receptor-meditated long-term depression
DHPG: (*S*)-3,5-Dihydroxyphenylglycine
SUnSET: Surface sensing of translation
S1: Stranger 1
E: Empty cage
S2: Stranger 2
EMX1: Empty spiracles homeobox 1
PV: Parvalbumin
SST: Somatostatin
LAMA1: Laminin
4EHP-eKO: Excitatory neuron-specific 4EHP knockout
LTD: Long-term depression
USVs: Ultrasonic vocalizations
p-ERK: Phospho-extracellular-signal-regulated kinase
DUSP: Dual-specificity phosphatase
MEFs: Mouse embryonic fibroblasts
HEK: Human embryonic kidney
FXS: Fragile X syndrome
vTRAP: Viral translating ribosome affinity purification
AAV: Adeno-associated virus
TE: Translational efficiency
