# Supplementary figures and images for: The eIF4E homolog 4EHP (eIF4E2) regulates hippocampal long-term depression and impacts social behavior
Source: Mol Autism. 2020 Nov 23;11:92. doi: 10.1186/s13229-020-00394-7 (PMC7682028; doi:10.1186/s13229-020-00394-7)

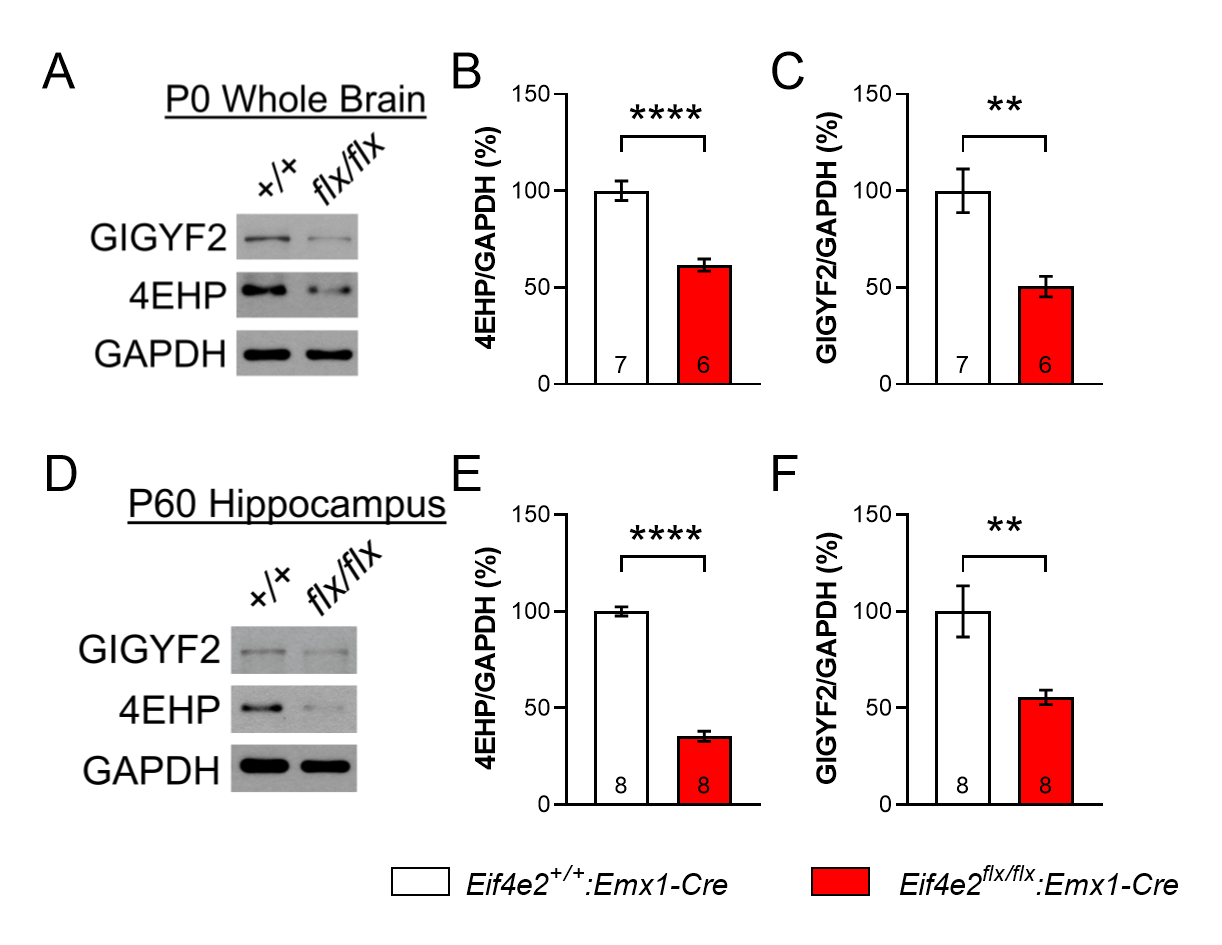

Supplement: Supplementary file 2 — Additional file 2. Figure 1: Codeletion of 4EHP and GIGYF2 occurs as early as P0 in the brain of 4EHP-eKO mice. A Western blot analysis of GIGYF2 and 4EHP levels in P0 whole brain from 4EHP-WT (+/+) versus 4EHP-eKO (flx/flx) mice. GAPDH was used as loading control. Band C Quantification of band intensity from A, presented as percent control. D Western blot analysis of GIGYF2 and 4EHP levels in P60 hippocampus from 4EHP-WT (+/+) versus 4EHP-eKO (flx/flx) mice. GAPDH was used as loading control. E and F Quantification of band intensity from D, presented as percent control. Data are presented as mean ± s.e.m.; **p<0.01, ****p<0.0001; calculated by unpaired t-test. Sample size is located within bar graphs. [file 13229_2020_394_MOESM2_ESM.tif]

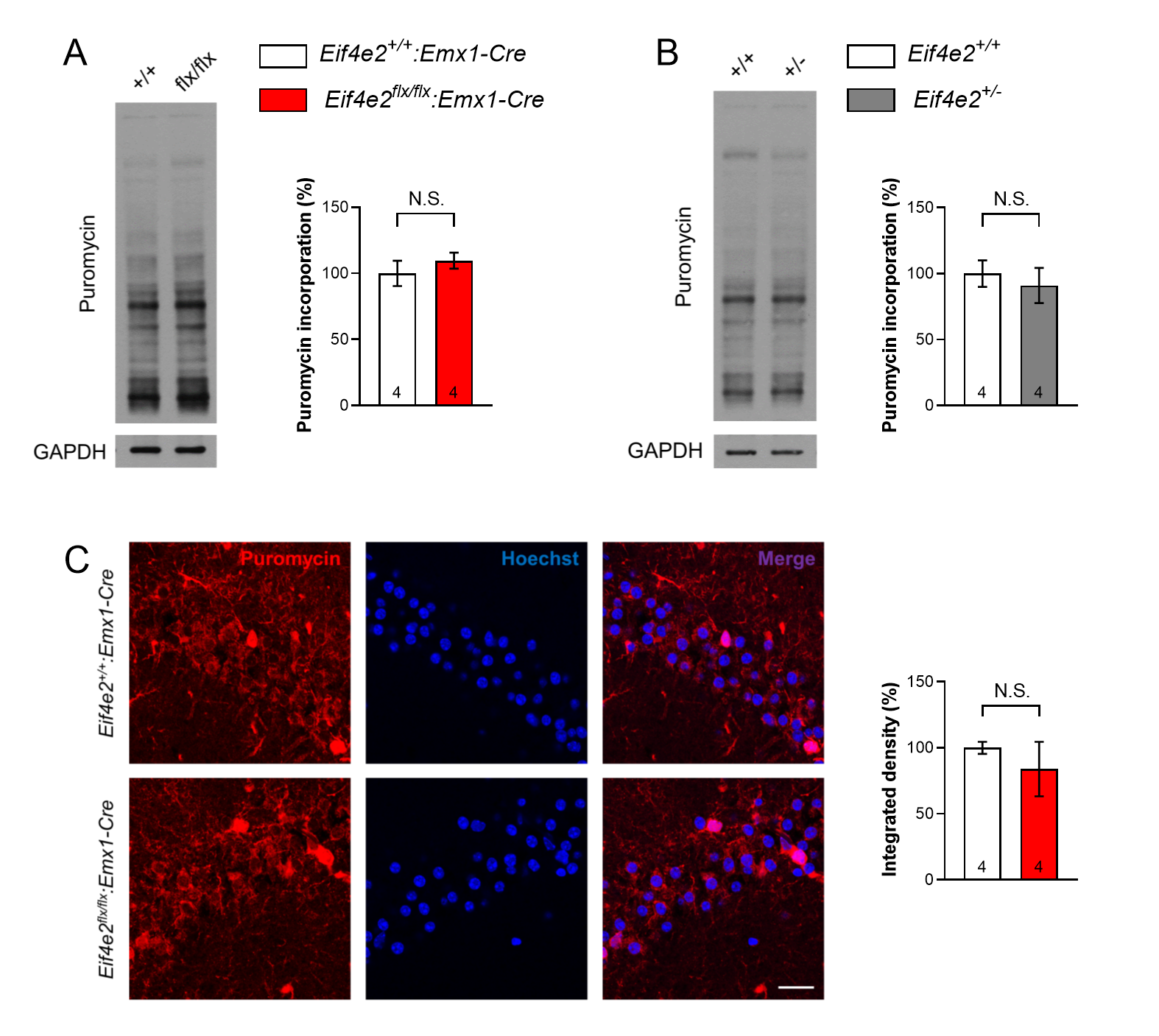

Supplement: Supplementary file 3 — Additional file 3. Figure 2: Analysis of global protein synthesis. A Puromycin incorporation into hippocampal slices from 4EHP-WT and 4EHP-eKO mice measured by western blot (left panel) and quantification (right panel) normalized to GAPDH. B Puromycin incorporation into hippocampal slices from 4EHP+/+ and 4EHP+/- mice measured by western blot (left panel) and quantification (right panel) normalized to GAPDH. C Puromycin incorporation into hippocampal slices from 4EHP-WT and 4EHP-eKO mice measured by immunofluorescence (left panel) and quantification (right panel). Puromycin staining is colored in red and Hoechst-stained nucleus in blue. Quantification of puromycin integrated density was performed on whole image using image J. Scale bar represents 20 µm. Data are presented as mean ± s.e.m.; N.S., not significant; calculated by unpaired t-test. Sample size is located within bar graphs. [file 13229_2020_394_MOESM3_ESM.tif]

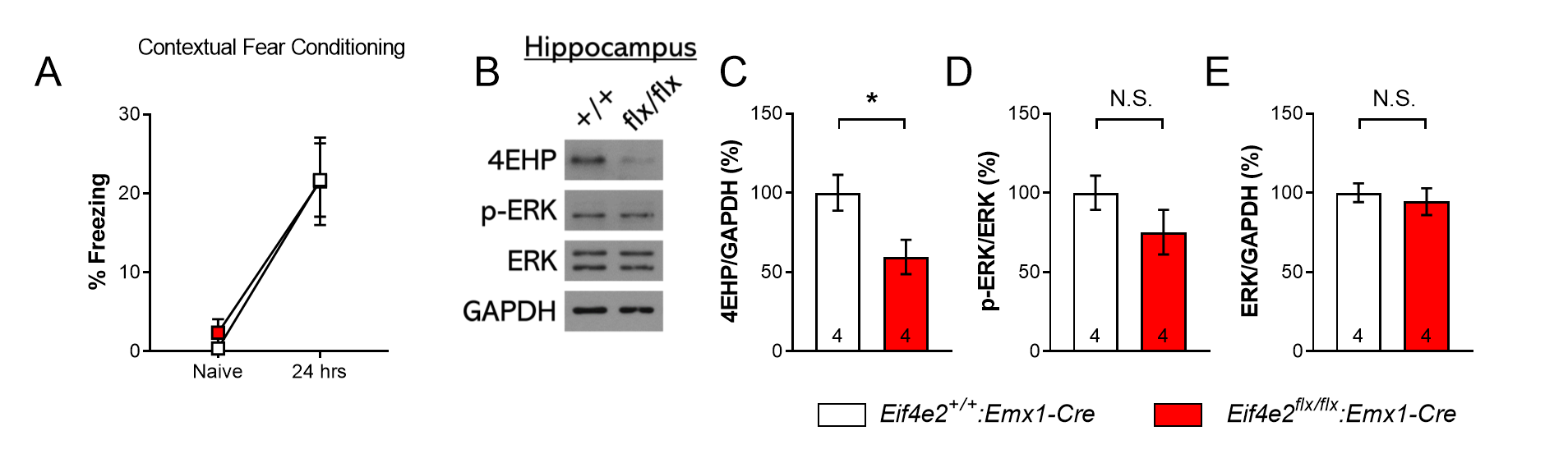

Supplement: Supplementary file 4 — Additional file 4. Figure 3: Analysis of long-term contextual fear memory and p-ERK. A Mice were placed into a soundproof box (context) with an electric grid floor. Freezing time was recorded for 2 min (Naïve) before receiving a mild foot shock (0.7 mA, 1 sec). Mice were placed back in the box after 24 hr and freezing behavior recorded, n=11 (4EHP-WT), n=9 (4EHP-eKO). B Western blot analysis of ERK activation (p-ERK) in the hippocampus of 4EHP-eKO versus 4EHP-WT mice. C Quantification of 4EHP normalized to GAPDH. D Quantification of p-ERK normalized to total ERK. E Quantification of total ERK normalized to GAPDH. Data are presented as mean ± s.e.m.; *p<0.05, N.S., not significant; calculated by unpaired t-test. Sample size is located within bar graphs. [file 13229_2020_394_MOESM4_ESM.tif]
